# Comparison of Three Real-Time PCR Assays for the Detection of *Cyclospora cayetanensis* in Stool Samples Targeting the 18S rRNA Gene and the *hsp70* Gene

**DOI:** 10.3390/pathogens11020165

**Published:** 2022-01-26

**Authors:** Felix Weinreich, Andreas Hahn, Kirsten Alexandra Eberhardt, Torsten Feldt, Fred Stephen Sarfo, Veronica Di Cristanziano, Hagen Frickmann, Ulrike Loderstädt

**Affiliations:** 1 Department of Microbiology and Hospital Hygiene, Bundeswehr Hospital Hamburg, 20359 Hamburg, Germany; felixweinreich@bundeswehr.org (F.W.); frickmann@bnitm.de (H.F.); 2 Department of Medical Microbiology, Virology and Hygiene, University Medicine Rostock, 18057 Rostock, Germany; andreas.hahn@uni-rostock.de; 3 Department of Tropical Medicine, Bernhard Nocht Institute for Tropical Medicine & I. Department of Medicine, University Medical Center Hamburg-Eppendorf, 20359 Hamburg, Germany; k.eberhardt@bnitm.de; 4 Department of Gastroenterology, Hepatology and Infectious Diseases, University Medical Center Düsseldorf, 40225 Düsseldorf, Germany; torsten.feldt@med.uni-duesseldorf.de; 5 Department of Medicine, Kwame Nkrumah University of Science and Technology, Kumasi 00233, Ghana; fsarfo.chs@knust.edu.gh; 6 Institute of Virology, Faculty of Medicine and University Hospital of Cologne, University of Cologne, 50935 Cologne, Germany; veronica.di-cristanziano@uk-koeln.de; 7 Department of Hospital Hygiene & Infectious Diseases, University Medicine Göttingen, 37075 Göttingen, Germany

**Keywords:** *Cyclospora cayetanensis*, stool, real-time PCR, diagnosis, test comparison, evaluation, validation, latent class analysis, without reference standard

## Abstract

Diagnostic real-time PCR for the detection of *Cyclospora cayetanensis* in human stool samples has been applied for two decades. However, recent comparative assessments between in-house and commercial assays suggested room for improvement regarding the agreement of positive signals of the applied real-time PCRs. In order to assess the effect of the choice of the target sequence, 3 inhouse real time PCR assays targeting the 18S rRNA gene (*n* = 2, one of them later referred to as SSU rRNA gene assay to avoid confusion) and the *hsp70* gene of *C. cayetanensis* were compared in a head-to-head comparison with 905 samples with high pretest probability for *C. cayetanensis* infections from Ghanaian HIV patients in a test comparison without a reference standard. Only slight agreement kappa of 0.095 was observed. In the assays targeting the SSU rRNA gene, the 18S rRNA gene, and *hsp70*, positive signals were recorded in 63, 45, and 0 instances, respectively, with latent class analysis-based estimation of sensitivity of 32.2%, 23.3%, 0% as well as of specificity of 99.7%, 99.9% and 100%, respectively. High cycle threshold values with an average of about 35 indicated low quantities of target DNA in the samples with similar Ct values in concordantly and discordantly positive samples. In conclusion, the study suggested target-gene-specific differences in the diagnostic accuracy of real-time PCR-based diagnosis of *C. cayetanensis* as well as an ongoing need for further standardization of this diagnostic approach.

## 1. Introduction

*Cyclospora cayetanensis* are coccidian parasites causing enteric disease in human patients following fecal-oral transmission [1,2] with an obligatory environmental sporulation step in water or soil [1]. Consequently, cyclosporiasis is correlated both with environmental exposition [3,4] and with increased occurrence in the rainy season [5,6]. Clinically apparent disease resulting from infections of the upper small intestinal tract is usually self-limiting in the immunocompetent host [7], while complications such as chronic diarrhea, villous atrophy and crypt hyperplasia have been described from immunodeficient patients [1,2,5,8,9].

As typical for fecal-orally transmitted pathogens, prevalence is particularly high in resource-limited settings where established hygiene regimens leave room for improvement as well as in travelers returning from such settings [1,10,11,12,13,14,15,16,17].

While the diagnosis of *C. cayetanensis* has traditionally been based on microscopy [2], usually following acid-fast staining or alternative approaches to increase visibility within stool specimens as well as enrichment steps to increase sensitivity [2,18,19,20,21], both in-house and commercial molecular diagnostic assays have been described in the meantime [5,22,23,24,25,26]. In recent comparisons of commercial and in-house real-time PCR assays, however, there was a surprisingly low agreement between different molecular *C. cayetanensis*-specific assays [25,26]. Interestingly, this finding was accompanied by a generally higher sensitivity of real-time PCR compared to microscopy [25,27]. The reasons for the observed mismatching between the recorded positive results in the commercial and the in-house real-time PCR assays [25,26] remained unclear. As the applied oligonucleotides are usually not published for commercial molecular diagnostic assays, it could not be checked whether or not different target sequences of the real-time PCR assays could be the reason for the observed discrepancy [25,26]. As the choice of the target sequence is of critical importance for the reliability of a diagnostic real-time PCR assay [28,29], it is likely that different target sequences as previously reported for *C. cayetanensis* [25,26,30,31,32,33] may have played a role.

In order to further investigate a likely target sequence dependence of inconsistent *C. cayetanensis* real-time PCR results, 3 different real-time PCR assays targeting the 18S ribosomal RNA (18S rRNA) gene (*n* = 2, one of them later referred to as the small subunit ribosomal RNA (SSU rRNA) gene assay to make the discrimination easier) and the heat shock protein 70 *(hsp70*) gene [25,26,30,31,32] were compared in a head-to-head comparison without a reference standard by applying a latent class analysis-(LCA-) based assessment [28,34]. The target genes were chosen for the comparisons as they are frequently applied in diagnostic *C. cayetanensis* assays [22,23,24,25,26,27,30,31,32], so their comparative assessment should be of interest for both test developers and clinical microbiologists interpreting the diagnostic results. To ensure a sufficient pre-test probability associated with enough positive test results for a meaningful investigation, residual sample materials from study participants from resource-limited settings with increased risk of *C. cayetanensis* infections [11,12,14,15,16] were chosen for the test comparison without a reference standard.

## 2. Materials and Methods

### 2.1. Residual Sample Collection and Nucleic Acid Extraction

A total of 905 nucleic acid extractions from stool samples collected from Ghanaian HIV patients (*n* = 905) [35,36] were used for this study. The samples had already been successfully applied for comparative evaluations of real-time PCR assays targeting either microsporidia [37] or other coccidian parasites [38,39], so a high likelihood of a relevant proportion of samples positive for various parasites could be assumed for the assessment. Assessments for *C. cayetanensis* had not been performed so far with these samples prior to this study.

### 2.2. Applied In-House Real-Time PCRs

In a head-to-head comparison without a reference standard, three real-time PCR assays targeting the SSU rRNA gene, the 18S rRNA gene and the *hsp70* gene (Table 1) were run on RotorGene Q cyclers (Qiagen, Hilden, Germany) or magnetic induction cyclers (MIC, Bio Molecular Systems Ltd., London, UK) as described [30,31,32] with minor modifications. All protocols had been established on both cycler models in the same laboratory in parallel and showed comparable performance characteristics on both. In detail, the Qiagen HotStar Taq master mix (Qiagen, Hilden, Germany) at the standard concentration as recommended by the manufacturer with a final Mg^2+^ concentration (Qiagen, Hilden, Germany) of 5 mM and 0.05 ng/mL bovine serum albumin (BSA; Carl Roth, Karlsruhe, Germany) was applied in 20 µL total reaction volumes containing 2 µL sample eluate each as decided in the course of the in-house optimization of the protocols. For the target genes SSU rRNA, 18S rRNA and *hsp70*, forward primer concentrations were 1 pmol/µL, 0.5 pmol/µL and 1.3 pmol/µL, reverse primer concentrations 2.0 pmol/µL, 0.5 pmol/µL and 1.3 pmol/µL, respectively, and probe concentrations 2.0 pmol/µL, 0.5 pmol/µL and 1.3 pmol/µL, respectively. The run conditions comprised an initial activation step at 95 °C for 15 min followed by 55 cycles comprising denaturation at 95 °C for 15 s, annealing for 30 s starting at 67 °C with a touchdown of 0.5 °C per cycle for 13 cycles, and amplification at 72 °C for 30 s each with final cooling down to 40 °C for 20 s. PCR inhibition was controlled with an inhibition control real-time PCR targeting a phocid herpes virus (PhHV) DNA sequence was described elsewhere [40]. Inhibited samples with positive *C. cayetanensis*-specific signals were excluded from the diagnostic accuracy assessment. All PCRs were run with a plasmid-based positive control (see Table A1 in Appendix A) and a PCR water-based negative control sample. As assessed with a dilution series of the positive control plasmid and calculated with the software SciencePrimer.com (http://scienceprimer.com/copy-number-calculator-for-realtime-pcr, last accessed on 23 December 2021), the limits of detection for the assays were less than 10 copies per µL eluate for the SSU rRNA and 18S rRNA PCRs. For the *hsp70* gene, a slightly higher limit of detection of 31 copies per µL was calculated.

### 2.3. Statistical Assessment

Latent class analysis (LCA) [28,34] was applied for the test comparison without a reference standard in order to calculate sensitivity and specificity of the applied test assays as well as for an accuracy-adjusted prevalence estimation. LCA is a variant of structural equation models which aims at estimating latent non-observed variables as the actual disease status over observed variables, e.g., the results of diagnostic test assays. Concordance according to Fleiss’ kappa was calculated and interpreted as described elsewhere [41]. In more detail, Fleiss’ kappa indicated the agreement between the qualitative results of the real-time PCRs with the strata poor (below 0.00), slight (0.00–0.20), fair (0.21–0.40), moderate (0.41–0.60), substantial (0.61–0.80), and almost perfect (0.81–1.00) as defined previously [41]. The cycle threshold (Ct) values of the real-time PCRs were descriptively compared. The statistical assessments were performed using the software Stata/IC 15.1 for Mac 64-bit Intel (College Station, TX, USA).

## 3. Results

From the 905 assessed samples of Ghanaian HIV patients, 33 were excluded from the further diagnostic accuracy estimations due to recorded sample inhibition as indicated by the PhHV-specific inhibition control PCR. Of note, those excluded samples comprised 1 sample positive in the SSU rRNA gene PCR and another sample positive in the 18S rRNA gene PCR.

In the non-inhibited samples, the recorded cycle threshold (Ct) values for the PhHV-DNA-based inhibition control target showed a mean value (±standard deviation SD) of 33.9 (±3.0) and a median (minimum, maximum) of 33.3 (23.1, 48.0). In total, recorded detections of the targeted *C. cayetanensis* sequences among the 872 included samples succeeded in 62 (7.1%) cases for the SSU rRNA gene, in 44 (5.1%) cases for the 18S rRNA gene, and in 0 (0%) cases for the *hsp70* gene. Thereby, 14 samples were indicated as positive by both SSU rRNA gene PCR and 18S rRNA gene PCR. LCA-based diagnostic accuracy-adjusted prevalence estimation resulted in a prevalence of 21.4% (14.0%, 31.4%) in the assessed population of Ghanaian HIV patients.

Agreement kappa over the 3 different compared assays was slight according to the interpretation standards as suggested by Landis and Koch [41]. Sensitivity as calculated by applying LCA was 32.2% for the SSU rRNA gene PCR, 23.3% for the 18S rRNA gene PCR and 0.0% for the *hsp70* PCR. The specificity values were 99.7% for the SSU rRNA gene PCR, 99.9% for the 18S rRNA gene PCR and 100% for the *hsp70* PCR as estimated by LCA. Due to the low agreement, no 0.95 confidence intervals could be calculated for these estimated specificity values, implying that their 0.95 confidence intervals could theoretically cover the full spectrum from 0% to 100%. Details including estimable confidence intervals are shown in Table 2, a cross table indicating the matches and mismatches among the 872 included samples is presented in Table 3.

Descriptive assessment of the recorded cycle threshold (Ct) values indicated predominantly high values of 35 cycles and more. Further, the differences between the SSU rRNA gene PCR and the 18S rRNA gene PCR were negligible with considerably overlapping confidence intervals. Focusing on the 14 samples positive in SSU rRNA gene PCR and 18S rRNA gene PCR, of note, the average and median Ct values were in the same range. Details are provided in Table 4.

## 4. Discussion

The study was performed to further investigate the recently observed phenomenon of considerable discordance of positive signals in in-house and commercial real-time PCR assays targeting *C. cayetanensis* [25,26]. In detail, slight agreement [41] with a kappa value of 0.184 (−0.064, 0.431) had been recorded in a comparison of the same in-house SSU rRNA gene PCR as applied in the present study and the LightMix Modular *Cyclospora* assay (TIB MOLBIOL, Berlin, Germany) targeting the 18S rRNA gene of *Cyclospora cayetanensis* in a test comparison without a reference standard with residual stool sample materials with a high pretest probability but without microscopic pre-characterization [26]. In another similar study comparing this in-house SSU rRNA gene assay with both microscopy and the *Cyclospora cayetanensis*-specific real-time PCR within the commercial Allplex GI panel 4 (SeeGene, Seoul, Korea), for which the molecular target is not publicly available, with residual stool sample materials from tropical travel returnees, only moderate agreement with a kappa value of 0.432 (0.309, 0.548) had been observed as well [25]. Low total numbers of *C. cayetanensis* detections in both previous studies made the interpretation of these findings challenging. However, the repeated observation of a likely agreement problem triggered the present study on a potential influence of commonly applied target genes on the diagnostic accuracy of the assays. Again, the study was conducted as a test comparison without a reference standard with residual samples with a high pre-test probability but without microscopic characterization, which were available from a previous epidemiological investigation with HIV-positive Ghanaian patients [35,36].

The observed slight agreement between SSU rRNA gene-specific, 18S rRNA gene-specific and *hsp70*-specific real-time PCR with a kappa of 0.095 (0.045, 0.164) in the present study is within the confidence interval of the kappa of 0.184 (−0.064, 0.431) as recorded in the previous comparison of in-house SSU rRNA gene PCR and commercial 18S rRNA gene PCR [26]. Of note, *hsp70* PCR did not show positive signals at all and so, the agreement of positive signals within the present study resulted from concordantly positive signals between the SSU rRNA gene PCR and the 18S rRNA gene PCR alone. This additional finding confirms the suspicion arising from the previous assessments [25,26] that the discordance between positive *C. cayetanensis* real-time PCR results is likely to be relevantly influenced by the choice of the target sequence among the commonly used real-time PCR targets for the diagnostic detection of *C. cayetanensis* [5,22,23,24,25,26,27,30,31,32,33]. This finding may seem trivial at first glance but is nevertheless in contrast to the promising findings of previous evaluation studies for the chosen *C. cayetanensis*-specific target genes [30,31,32], according to which higher diagnostic reliability and thus better agreement should have been expected.

In addition, the details as observed in this study deserve some further consideration as well. First of all, it is noteworthy that the average cycle threshold (Ct) values were high in all positive samples, indicating the abundance of low quantities of target DNA. This implies that a relevant proportion of samples contained target DNA amounts close to the diagnostic detection limit and thus in a range, in which PCR-amplification events become stochastic. Reduced agreement of positive results due to stochastic amplification at the diagnostic detection limit is thus an explanatory hypothesis to be considered, while unequal meta-structures due to specificity limitations or insufficient sequence conservation of the PCR targets would be an alternative explanation. The high specificity values as calculated by LCA are in contradiction to this latter alternative explanation. However, those calculations need to be interpreted with care, as the low agreement of the positive results made a calculation of the confidence intervals impossible. For the same reason, LCA-estimated sensitivity values were very low for all assessed *C. cayetanensis*-specific real-time PCR assays. Second, the averaged Ct values in concordantly positive samples in SSU rRNA gene PCR and 18S rRNA gene PCR were not lower than in discordantly positive samples, indicating that the sole effect of the target DNA amount on the agreement is probably not the only explanation for the observed high discordance rate. Third, there was moderate variance within the measured inhibition control PCR cycle threshold (Ct) values, however, such partial inhibition should have affected all *C. cayetanensis*-specific real-time PCR in a similar way. However, the recorded partial inhibition phenomena suggest that the true «real-world» diagnostic detection limits were most likely higher than the recorded detection limits as measured with positive control plasmid DNA under standardized conditions. This may explain why *hsp70*-specific real-time PCR, which showed comparably higher detection limits even under optimized and standardized conditions, missed all samples in this assessment. The fact that low target DNA quantities were indicated by the recorded Ct values in the Ghanaian samples further supports this explanatory hypothesis.

Differences in the number of gene copies are another likely reason for the comparably poor sensitivity of the *hsp70*-specific real-time PCR. As a single copy-gene, *hsp70* is more likely to be missed than ribosomal multi-copy genes if the target DNA concentration is close to the diagnostic detection limit. In contrast, 18 copies of the 18S rRNA gene have been described for *C. cayetanensis* [42], which may at least partially explain the recorded sensitivity difference.

As an interesting side finding, LCA-based diagnostic accuracy-adjusted prevalence estimation suggested a prevalence of 21.4% for *C. cayetanensis* in the assessed study population of HIV-positive Ghanaian patients. This is surprisingly high, compared to 6.0% for *Cryptosporidium* spp. and 3.2% for *Cystoisospora belli* as previously reported for this population [38,39]. While an association of increased prevalence of the coccidian parasites *Cryptosporidium* spp. and *C. belli* with HIV infection is considered as sufficiently confirmed, such an association has been reported to be much less well established for *C. cayetanesis* [43]. The data presented in this study are in contradiction to this doctrine and further suggest that insufficient diagnostic sensitivity might have contributed to an underestimation of *C. cayetanensis* prevalence in HIV-infected individuals in previous epidemiologic assessments [43].

The study has a number of limitations. First, the DNA samples were old and characterization by microscopy was lacking, which further reduced their value as reference materials for diagnostic accuracy assessments. However, adequate sample storage at −80 °C makes quantitatively relevant DNA degradation less likely and rare diagnoses of cyclosporiasis make excellently characterized *C. cayetanasis*-positive reference samples a rare resource. So, compromises regarding the choice of the samples were required and LCA helped to a least control the associated risk of bias in this test comparison without a reference standard. Second, sequencing of the PCR amplicons would have been useful to confirm or exclude specificity of individual positive real-time PCR signals. Unfortunately, funding-associated restrictions did not allow this cost-intensive confirmatory procedure, so biostatistical estimation of diagnostic accuracy was the method of choice. Third, the assessed in-house real-time PCR assays represent only a subset of described assays targeting commonly used target genes as described elsewhere [25,26,30,31,32,33]. So, they are not necessarily representative of the target genes and differing results might have resulted from a different choice of assays. Accordingly, the study just comprises a proof-of-principle assessment on target-gene dependence of real-time PCR based diagnosis of *C. cayetanensis*. Fourth, due to the demands by the ethical clearance for this evaluation study to be performed with anonymized diagnostic residual sample materials, no clinical data can be presented, which is an admitted deviation from the STARD (Standards for Reporting Diagnostic Accuracy) criteria [44]. Fifth, lacking availability of standardized concentrations of well-defined oocysts did not allow spiking experiments with the parasites but just the use of positive control plasmids for the assessments of the detection limits of the assays. Plasmid-based detection limits have to be interpreted with care as they do not include the releasing step of the target DNA. So, they are likely to underestimate the true detection limit in the diagnostic situation and can only be considered as an approximation.

## 5. Conclusions

In spite of the above-mentioned limitations, the study confirms previous findings on low agreement between positive results of real-time PCR assays targeting *C. cayetanensis* in human stool samples [25,26] and suggests an association of this poor concordance with the choice of the target genes. In spite of seemingly excellent specificity in contrast to relatively poor sensitivity of the tested *C. cayetanensis*-specific real-time PCR assays in LCA, the question on the actual accuracy of individual positive real-time PCR signals remains unresolved. Therefore, it is recommended to confirm a positive real-time PCR result indicating the abundance of *C. cayetanensis* DNA in a stool sample by at least another diagnostic assay before a sample should be considered as a confirmed positive reference material for test evaluation and validation purposes. In addition, the results of the study call for ongoing standardization of molecular diagnostic assays targeting *C. cayetanensis*.

## Figures and Tables

**Table 1:** Applied oligonucleotide sequences of the compared *C. cayetanensis*-specific real-time PCR assays [30,31,32].

Forward Primer Name	Forward Primer Sequence	Reverse Primer Name	Reverse Primer Sequence	Probe Name	Probe Sequence
SSU rRNA gene PCR according to Verweij et al. [30]					
Cyclo250F	5′-TAGTAACCGAACGGATCGCATT-3′	Cyclo350R	5′-AATGCCACGGTAGGCCAATA-3′	Cyclo281T	5′-CCGGCGATAGATCATTCAAGTTTCTGACC-3′
18S rRNA gene PCR according to Varma et al. [31]					
VarmaF	5′-TGAACTCATTGGACTGACCAGC-3′	VarmaR	5′-ACTTTTGCATCCTTTAGAGGGCT-3′	VarmaP	5′-TTCGCGGAGCTGGTCGGAAAGTTG-3′
*hsp70* gene PCR according to Shields et al. [32]					
HMPr36	5′-GGGTAAGCCACTTATTGA-3′	HMPr40	5′-GCCTCCTTAACTTCTTTG-3′	HMPro53	5′-CCTTCATCTTCACCAGCACCA-3′

**Table 2:** Agreement kappa between the compared real-time PCR assays as well as sensitivity, specificity and accuracy-adjusted prevalence as calculated with latent class analysis (LCA). Samples showing inhibition in the inhibition control PCR were excluded from the assessment, resulting in a total of 872 included samples.

Assay	*n*	Positives (%)	Sensitivity (0.95 CI)	Specificity (0.95 CI)	Kappa (0.95 CI)
SSU rRNA gene PCR according to Verweij et al. [30]	872	62 (7.11)	0.322 (0.200, 0.473)	0.997 (n.e.)	0.095 (0.045, 0.164)
18S rRNA gene PCR according to Varma et al. [31]	872	44 (5.05)	0.233 (0.142, 0.357)	0.999 (n.e.)	
*hsp70* PCR according to Shields et al. [32]	872	0	0	1 (n.e.)	
Prevalence (0.95 CI)	0.214 (0.140, 0.314)				

0.95 CI = 95%-confidence intervals. *n* = numbers. n.e. = non-estimable.

**Table 3:** Cross-table detailing matches and mismatches between the SSU rRNA gene and the 18S rRNA gene PCR assays. Samples showing inhibition in the inhibition control PCR were excluded from the assessment, resulting in a total of 872 included samples. The *hsp70* PCR was not included in the cross-table, as it did not show positive results at all and so, it was just discordant with 62 positive signals in the SSU rRNA gene PCR and with 44 positive signals in the 18S rRNA gene PCR.

		SSU rRNA Gene PCR according to Verweij et al. [30]	
		Negative	Positive
**18S rRNA gene PCR according to Varma et al. [31]**	negative	780	48
	positive	30	14

**Table 4:** Recorded cycle threshold (Ct) values of the real-time PCR assays. All positive test results among the 905 assessed samples were included, also the 2 samples with positive PCR signals which had been excluded from diagnostic accuracy assessment due to sample inhibition as stated above.

Assay	*n*	Mean (SD)	Median (Min, Max)
SSU rRNA gene PCR according to Verweij et al. [30]	63	34.4 (±4.39)	35.04 (23.78, 43.77)
SSU rRNA gene PCR according to Verweij et al. [30] for samples also positive in the 18S rRNA gene PCR	14	35.43 (±4.55)	34.92 (23.78, 42.67)
18S rRNA gene PCR according to Varma et al. [31]	45	36.92 (±3.26)	36.53 (30.89, 46.90)
18S rRNA gene PCR according to Varma et al. [31] for samples also positive in the SSU rRNA gene PCR	14	36.40 (±3.19)	35.87 (33.60, 46.90)
*hsp70* PCR according to Shields et al. [32]	0	n.a.	n.a.

*n* = numbers. SD = standard deviation. Min = minimum. Max = maximum. n.a. = not applicable.

## Data Availability

All relevant data are provided within the manuscript. Raw data can be provided on reasonable request.

## Appendix A

**Table A1:** Sequence inserts for the positive control plasmid, which was based on a pEX-A128 vector backbone.

Positive Control Insert Based on *C. cayetanensis* Sequences according to the NCBI GenBank Accession Numbers CSU40261, AF111183, HQ110607.
5′-GAATTCGATTCATAGTAACCGAACGGATCGCATTTGGCTTTAGCCGGCGATAGATCATTCAAGTTTCTGACCT ATCAGCTTTCGACGGTAGGGTATTGGCCTACCGTGGCATTGACGGGGAATTCGAGGGTCCTGTGAACTCATTG GACTGACCAGCTGTGCTTCGCGGAGCTGGTCGGAAAGTTGCGTAAATAGAGCCCTCTAAAGGATGCAAAAGT CGTAACACGGGAATTCCTTTCTTCCGGTAGCCTTCCGCGCTTCGCTGCGTGCGTTGGTGTTCCGGAACTTTTAC TTTGAGAAAAATAGAGTGTTTCAAGCAGGCTTGTCGCCCTGAATACTGCAGCATGGAATAATAAGATAGGAC CTTGGTTCTATTTTGTTGGTTTCTAGGACCGAGGTAATGATTAATAGGGACAGTTGGGGGCATTCGTATTTAAC TGTCAGAGGTGAAATTCTTAGATTTGTTAAAGACGAACTACTGCGAAAGCATTTGCCAAGGATGTTTTCATTA ATCAAGAACGACAGTAGGGGGTTTGAAGACGATTAGATACCGAATTCTGAGTGTGCATCGTGATGGGGATAG ATTATTGCAATTATTAATCTTCAACGAGGAATGCCTAGTAGGCGCAAGTCAACAGCTTGCGCCGATTACGTCC CTGCCCCTTGTACACACCGCCCGTCGCTGCAACCGATCGGAGGGTCCTGTGAACTCATTGGACTGACCAGCTG TGCTTCGCGGAGCTGGTCGGAAAGTTGCGTAAATAGAGCCCTCTAAAGGGAATTCCTGGAAGCGGGGGTAAG CCACTTATTGAAGTGAACTACCAAGGTGCTACGAAGACTTTTCATCCGGAGGAAATTTCCGCCATGGTGCTGG TGAAGATGAAGGAAATTGCCGAGTCGTTCGTTGGCAAAGAAGTTAAGGAGGCCGTTATTACAGAATTCAATT TTGGGCGAATCACAGATTGAATCTGATGATACAGCAACATTTTTTATGTGTCCGCCACCATCTGGATCAACGT TGGTACGTTTGGAACCGCCTCGGGCGAATTC-3′
